# Configuration method of BESS in the wind farm and photovoltaic plant considering active and reactive power coordinated optimization

**DOI:** 10.1371/journal.pone.0257885

**Published:** 2021-10-13

**Authors:** Li Zhang, Weigang Huang, Peng Kang, Linfeng Zeng, Yong Zheng, Feng Zheng

**Affiliations:** Chongqing Fuling Electric Power Industry Co., Ltd, Chongqing, China; University of Science and Technology of China, CHINA

## Abstract

To promote the coordinated development between renewable energy and the distribution network, a capacity allocation model of battery energy storage systems (BESS) is proposed to achieve the coordinated optimization for active and reactive power flow, which can reduce the voltage deviation and improve the absorptive capacity for renewable energy. In addition, BESS with four-quadrant operation characteristics, on-load tap changer, and capacitor banks are treated as flexible devices to improve the adaptability for renewable energy fluctuations. In view of the uncertainties of renewable energy caused by the inaccuracy of historical sample data, a set of extreme scenarios with the characteristics of temporal and spatial correlation are considered to obtain a robust BESS configuration decision. The big-M approach and the second-order conic relaxation technique are utilized to convert the BESS capacity allocation model into a mixed-integer linear programming problem. Finally, the IEEE 33-node distribution system is taken as an example to verify the effectiveness of the proposed method.

## Introduction

In recent years, the penetration of renewable energy has increased rapidly to replace traditional fossil fuels due to its clean and renewable characteristics [1]. However, the uncertainty and intermittent characteristics of wind power and photovoltaic (PV) power can bring many challenges to the safety and stability of the distribution network operation [2]. In this regard, energy storage is considered as a highly flexible device, which has the potential to promote the coordinated development of renewable energy and distribution network [3]. The battery energy storage system (BESS) attracts more attention because of its characteristics of high energy density and fast operation. In ISO New England, 94 MW of the battery energy storage capacity has been proposed for deployment as of January 2016 [4]. The California Public Utilities Commission has mandated a merchant BESS procurement goal of 1325 MW by 2020 [5]. With the wide application of BESS in the distribution network, it is significant to obtain an optimal configuration scheme of BESS for achieving lots of benefits. In [6], the BESS is applied to enhance the power quality and operation stability of the distribution network. In addition, considering the performance of spatiotemporal arbitrage, the installation of BESS is conducive to reducing the cost of dealing with the intermittency of renewable resources [7]. In [8], suitable size and location of BESS are investigated from the reduction of power loss and the better utilization of wind. The authors of [9] through the deployment of BESS to improve voltage quality in the distributed system. However, not only the benefit for distribution system but also a sufficient return on investment should be considered, because the BESS is often deployed and installed by private investors. The techno-economic model of PV-battery [10] and lifecycle-based cost modeling of batteries [11] are reported to ensure the economic interest of BESS investors.

BESS has the advantage of improving both power quality and economical operation, which is a promising technology for achieving active and reactive power optimization. In [12], a combined problem formulation with embedded wind turbine (WT) generation and battery storage is proposed for active-reactive optimal power flow in distribution network. Active power optimization is regarded as a method for energy management to minimize the total operation costs in [13]. In addition, reactive power optimization is conducive to decreasing the voltage deviation and power loss. In [14], a robust optimization scheme of active and reactive power is developed by coordinating the on-load tap changer (OLTC), reactive power compensators such as capacitor banks (CBs), and energy storage system (ESS). Therefore, the optimal configuration for BESS containing coordinated dispatch of active and reactive power is worth exploring research.

Furthermore, the uncertainty of renewable energy should be considered to realize the more rigorous and accurate allocation decisions for BESS. In this regard, stochastic programming [15] and chance-constrained optimization [16] are introduced into the study of energy storage capacity planning. However, all of them usually depend on the probability distributions of wind power characteristics. These distributions may ignore extreme scenarios in the historical data, which cannot ensure the configuration scheme of BESS is robust for renewable energy scenarios. As for traditional robust optimization problem, the box-like uncertainty set might be considered too conservative [17], and the robust counterpart could be intractable if the problem contains integer variables and cone constraints.

The main contributions of this paper can be represented as follows:

1. A configuration model for BESS capacity in the wind farm and the photovoltaic plant is proposed to effectively raise generation accommodation levels, where the coordinated optimization framework for active and reactive power flow is developed. The BESS with the power four-quadrant operation strategies is coordinated with on-load tap changer and capacitor banks to improve the voltage quality and increase the economic benefit.
2. A set of data-driven extreme scenarios is employed to describe the uncertainties of renewable energy. Based on the scenarios, the decision obtained of BESS configuration is robust. It is worth mentioning that the implementation of this method does not depend on dual transformation, which had been proved in the literature [18].

## The optimal model for BESS configuration

### Objective function

The optimization allocation for BESS capacity originally formulated within the target years is modeled using a representative day. The optimization target includes two parts: (i) the costs of the BESS investment and operation; (ii) the costs of the distribution network operation. As a result, the objective function is as follows:

$$\min\left(C_{1}+C_{2}\right)$$ (1)

Here, *C*_1_, *C*_2_ are the total costs of BESS and distribution network, respectively.

The operation and investment costs of BESS are described via (2)-(5), and the constraints (6)-(9) belong to the description of system operation cost. The consumer-price inflation for investment can be denoted with a constraint (3). The system operation costs consist of the electricity cost of the transmission network (TN), the penalty cost of power loss, and the penalty cost of wind and photovoltaic power curtailment, which can be expressed as constraints (7), (8), and (9), respectively.


$$C_{1}=\frac{1}{365}\left(aC_{\mathrm{inv}}+C_{\mathrm{on}}\right)$$ (2)

$$a=\frac{\kappa {\left(1+\kappa \right)}^{\varsigma }}{{\left(1+\kappa \right)}^{\varsigma }-1}$$ (3)

$$C_{\mathrm{inv}}=\sum_{i=1}^{N}\left(\mu_{S}S_{i}^{\mathrm{BESS}}+\mu_{E}E_{i}^{\mathrm{BESS}}\right)$$ (4)

$$C_{\mathrm{on}}=\sum_{i=1}^{N}\left(\mu_{S}\mu_{m}S_{i}^{\mathrm{BESS}}\right)$$ (5)

$$C_{2}=\frac{1}{Ns}\left(C_{\mathrm{buy}}+C_{\mathrm{loss}}+C_{\text{cur}}\right)$$ (6)

$$C_{\mathrm{buy}}=\sum_{s=1}^{Ns}\sum_{t=1}^{T}c_{t}^{\text{TN}}P_{t,s}^{\text{TN}}$$ (7)

$$C_{\mathrm{loss}}=\sum_{s=1}^{Ns}\sum_{t=1}^{T}\sum_{ij\in \Omega^{DN}}c_{\mathrm{loss}}{\tilde{I}}_{ij,t,s}r_{ij}$$ (8)

$$C_{\text{cur}}=\sum_{s=1}^{Ns}\sum_{t=1}^{T}c_{\text{cur}}(P_{t,s}^{\text{WT,cur}}+P_{t,s}^{\text{PV,cur}})$$ (9)

Here, *C*_inv_, *C*_on_are the investment and operation cost of BESS, respectively; *a* is capital recovery factor; *κ* is annual discount rate; *ς* is the lifetime of BESS; *N* is the total number of BESS installed in distribution network; *μ*_*S*_, *μ*_*E*_ are unit power capacity price and unit energy capacity price, respectively; *μ*_*m*_ is the maintenance cost rate of BESS; $S_{i}^{\mathrm{BESS}}$, $E_{i}^{\mathrm{BESS}}$ are the apparent power capacity and energy capacity of BESS, respectively; *Ns* is the total number of extreme scenarios; *T* is total scheduling period; Ω^*DN*^ is the set of total branches; *C*_buy_, *C*_loss_, and *C*_cur_ are the electricity cost of the TN, the penalty cost of power loss, and the penalty cost of wind and photovoltaic power curtailed, respectively; $c_{t}^{\mathrm{TN}}$ is unit price for active power from the TN; *c*_loss_ is unit penalty price for power loss; *c*_cur_ is unit penalty price for wind and photovoltaic power curtailment, respectively; ${\tilde{I}}_{ij,t,s}$ is the current square of branch *ij* at time *t* scenario *s*; *r*_*ij*_ is the resistor of branch *ij*; $P_{t,s}^{\text{TN}}$ is active power from the TN at time *t* scenario *s*; $P_{i,s,t}^{\text{WT,cur}}$, $P_{i,s,t}^{\text{PV,cur}}$are wind and photovoltaic power curtailed for bus *i* at time *t* scenario *s*, respectively.

### The equipment coordinated operation constraints

1. The BESS constraints
  Investors can plan the capacity range of BESS installation in advance according to the historical records of actual load demand and scheduling arrangement. The apparent power and energy capacity of BESS installed in the distribution network should meet the following constraints:
$$\text{0}\leq S_{i}^{\mathrm{BESS}}\leq S_{i,\max}^{\mathrm{BESS}}$$ (10)
$$\text{0}\leq E_{i}^{\mathrm{BESS}}\leq E_{i,\max}^{\mathrm{BESS}}$$ (11)
  Here, $S_{i,\max}^{\mathrm{BESS}}$ and $E_{i,\max}^{\mathrm{BESS}}$ are the upper bounds of apparent power capacity and energy capacity of BESS at bus *i*, respectively.
  Apart from charging or discharging active power, the BESS can compensate reactive power and absorb external reactive power due to its flexible power four-quadrant operation strategies. As a result, the BESS operation constraints are as follows:
$$\{\begin{array}{c} {\left(\beta_{c,i,t,s}^{\mathrm{BESS}}P_{c,i,t,s}^{\mathrm{BESS}}\eta \right)}^{2}+{\left(Q_{i,t,s}^{\mathrm{BESS}}\right)}^{2}\leq {\left(S_{i}^{\mathrm{BESS}}\right)}^{2}\\ \begin{array}{l} {\left(\frac{\beta_{d,i,t,s}^{\mathrm{BESS}}P_{d,i,t,s}^{\mathrm{BESS}}}{\eta }\right)}^{2}+{\left(Q_{i,t,s}^{\mathrm{BESS}}\right)}^{2}\leq {\left(S_{i}^{\mathrm{BESS}}\right)}^{2}\\ \begin{array}{cc} 0\leq P_{c,i,t,s}^{\mathrm{BESS}}, & 0\leq P_{d,i,t,s}^{\mathrm{BESS}} \end{array} \end{array} \end{array}$$ (12)
  Here, $\beta_{c,i,t,s}^{\mathrm{BESS}}$, $\beta_{d,i,t,s}^{\mathrm{BESS}}$ are the binary charging and discharging decisions of BESS for bus *i* at time *t* scenario *s*, respectively; $P_{c,i,t,s}^{\mathrm{BESS}}$, $P_{d,i,t,s}^{\mathrm{BESS}}$ are the charge and discharge power of BESS for bus *i* at time *t* scenario *s*, respectively; $Q_{i,t,s}^{\mathrm{BESS}}$is the reactive power of BESS for bus *i* at time *t* scenario *s*; *η* is the round-trip efficiency of BESS.
  In the scheduling period, it is commonly recognized that the energy level in the storage unit at the initial time point should be equivalent to that at the final time point. In order to ensure the orderly charging and discharging of BESS in a day, the charging/discharging power and the energy level should meet the following constraints:
$$E_{i,t,s}^{\mathrm{BESS}}=E_{i,t-1,s}^{\mathrm{BESS}}+\beta_{c,i,t-1,s}^{\mathrm{BESS}}P_{c,i,t-1,s}^{\mathrm{BESS}}\eta -\beta_{d,i,t-1,s}^{\mathrm{BESS}}\frac{P_{d,i,t-1,s}^{\mathrm{BESS}}}{\eta }$$ (13)
$$\left(1-D\right)E_{i}^{\mathrm{BESS}}\leq E_{i,t,s}^{\mathrm{BESS}}\leq E_{i}^{\mathrm{BESS}}$$ (14)
$$E_{i,1,s}^{\mathrm{BESS}}=E_{i,T,s}^{\mathrm{BESS}}=\gamma E_{i}^{\mathrm{BESS}}$$ (15)
$$\beta_{c,i,t,s}^{\mathrm{BESS}}+\beta_{d,i,t,s}^{\mathrm{BESS}}\leq 1$$ (16)
  Here, *D* is the maximum discharge depth of BESS; *γ* is the energy level of BESS at the initial time point and final time point.
2. The OLTC constraints
$${\tilde{V}}_{i,t}=V_{0,t}^{2}\left(r_{0}^{2}+\sum_{\tau }\left(\Delta r_{\tau }^{2}x_{i,t,\tau }^{\mathrm{OLTC}}\right)\right)$$ (17)
$$\{\begin{array}{c} \sum_{\tau }\left(x_{i,t,\tau }^{\mathrm{OLTC}}-x_{i,t-1,\tau }^{\mathrm{OLTC}}\right)\geq {\bar{l}}_{i,t}-{\underline{l}}_{i,t}N^{\mathrm{OLTC}}\\ \sum_{\tau }\left(x_{i,t,\tau }^{\mathrm{OLTC}}-x_{i,t-1,\tau }^{\mathrm{OLTC}}\right)\geq {\bar{l}}_{i,t}N^{\mathrm{OLTC}}-{\underline{l}}_{i,t} \end{array}$$ (18)
$$\{\begin{array}{l} x_{i,t,\tau -1}^{\mathrm{OLTC}}\geq x_{i,t,\tau }^{\mathrm{OLTC}}\\ \sum_{t}\left({\bar{l}}_{i,t}+{\underline{l}}_{i,t}\right)\leq N_{\max}^{\mathrm{OLTC},\text{op}}\\ {\bar{l}}_{i,t}+{\underline{l}}_{i,t}\leq 1 \end{array}$$ (19)
  Here, *V*_0,*t*_ is the voltage of transformers in primary side; *r*_0_ is the lower bound of tap ratio for OLTC; $x_{i,t,\tau }^{\mathrm{OLTC}}$is a 0–1 dummy binary variable for bus *i* equipped with OLTC in step *τ* at time *t*; Δ*r* is the ratio difference of OLTC adjacent step; ${\bar{l}}_{i,t}\;\text{and}\;{\underline{l}}_{i,t}$ are the regulation status of OLTC bus *i* at time *t*; *N*^OLTC^ is the range of OLTC regulation; $N_{\max}^{\mathrm{OLTC},\text{op}}$ is the maximum regulation number of OLTC.
3. The CBs constraints
$$Q_{i,t}^{\text{CBs}}=\sum_{\upsilon }\left(\Delta Q_{\upsilon }^{\text{CBs}}x_{i,t,\upsilon }^{\text{CBs}}\right)$$ (20)
$$\{\begin{array}{c} \sum_{\upsilon }\left(x_{i,t,\upsilon }^{\text{CBs}}-x_{i,t-1,\upsilon }^{\text{CBs}}\right)\geq {\bar{\omega }}_{i,t}-{\underline{\omega }}_{i,t}N^{\text{CBs}}\\ \sum_{\upsilon }\left(x_{i,t,\upsilon }^{\text{CBs}}-x_{i,t-1,\upsilon }^{\text{CBs}}\right)\leq {\bar{\omega }}_{i,t}N^{\text{CBs}}-{\underline{\omega }}_{i,t} \end{array}$$ (21)
$$\{\begin{array}{l} x_{i,t,\upsilon -1}^{\text{CBs}}\geq x_{i,t,\upsilon }^{\text{CBs}}\\ \sum_{t}\left({\bar{\omega }}_{i,t}+{\underline{\omega }}_{i,t}\right)\leq N_{\max}^{\text{CBs},\text{op}}\\ {\bar{\omega }}_{i,t}+{\underline{\omega }}_{i,t}\leq 1 \end{array}$$ (22)
  Here, $\Delta Q_{\upsilon }^{\text{CBs}}$ is the reactive power of per unit CBs; $x_{i,t,\upsilon }^{\text{CBs}}$ is a 0–1 dummy variable for bus *i* with CBs connected in step *ν* at time *t*; ${\bar{\omega }}_{i,t}\;\text{and}\;{\underline{\omega }}_{i,t}$ are the regulation status of CB for bus *i* at time *t*; *N*^CBs^ is the range of CBs regulation; $N_{\max}^{\text{CBs},\text{op}}$ is the maximum regulation number of CBs.
4. The constraints of WT and PV
$$0\leq P_{i,t,s}^{\text{WT,cur}}\leq P_{i,t,s}^{\mathrm{WT}}$$ (23)
$$0\leq P_{i,t,s}^{\text{PV,cur}}\leq P_{i,t,s}^{\text{PV}}$$ (24)
$$Q_{\min}^{\text{WT}}\leq Q_{i,t,s}^{\text{WT}}\leq Q_{\max}^{\text{WT}}$$ (25)
  Here, $P_{i,t,s}^{\mathrm{WT}}$ and $Q_{i,t,s}^{\text{WT}}$ are the active and reactive power of wind generation for bus *i* at time *t* scenario *s*, respectively; $P_{i,t,s}^{\text{PV}}$ is photovoltaic power for bus *i* at time *t* scenario *s*; $Q_{\min}^{\text{WT}}$ and $Q_{\max}^{\text{WT}}$ are the lower and upper bounds of reactive power of wind generation.
5. The constraints of power from the TN
$$0\leq P_{t,s}^{\text{TN}}\leq P_{\max}^{\text{TN}}$$ (26)
$$0\leq Q_{t,s}^{\text{TN}}\leq Q_{\max}^{\text{TN}}$$ (27)
  Here, $P_{\max}^{\text{TN}}$ and $Q_{\max}^{\text{TN}}$ are the upper bounds of active and reactive power from TN, respectively; $Q_{t,s}^{\text{TN}}$ is the reactive power from the TN at time *t* scenario *s*.

### Branch flow model

For the radial distribution network, the power flow constraints can be expressed by the branch power flow equations as follows:

$$\{\begin{array}{c} \sum_{k\in \delta \left(j\right)}P_{jk}=\sum_{i\in \pi \left(j\right)}\left(P_{ij}-I_{ij}^{2}r_{ij}\right)+P_{j}\\ \sum_{k\in \delta \left(j\right)}Q_{jk}=\sum_{i\in \pi \left(j\right)}\left(Q_{ij}-I_{ij}^{2}x_{ij}\right)+Q_{j} \end{array}$$ (28)

$$V_{j}^{2}=V_{i}^{2}-2\left(r_{ij}P_{ij}+x_{ij}Q_{ij}\right)+\left(r_{ij}^{2}+x_{ij}^{2}\right)I_{ij}^{2}$$ (29)

$$I_{ij}^{2}=\frac{P_{ij}^{2}+Q_{ij}^{2}}{V_{i}^{2}}$$ (30)

Here, *δ*(*j*) and *π*(*j*) are the sets of the parent nodes and child nodes, respectively; *i*, *j*, and *k* are the indices of buses; *ij* and *jk* are the indices of branches; *P*_*j*_, *Q*_*j*_ are total active and reactive power injection at bus *j*, respectively; *P*_*ij*_, *Q*_*ij*_ are the active and reactive power flow of branch *ij*, respectively; *V*_*i*_ is the voltage at bus *i*; *I*_*ij*_ is the current of branch *ij*; *r*_*ij*_, *x*_*ij*_ are the resistance and reactance of branch *ij*, respectively.

The above branch power flow Eqs (28)–(30) contain nonlinear terms, which can be linearized as formulation (31). Furthermore, the Eq (30) can be reformulated standard second-order cone (SOC) constraint (32) by convex relaxation technology. [19]


$$\{\begin{array}{c} {\tilde{I}}_{ij}=I_{ij}^{2}\\ {\tilde{V}}_{i}=V_{i}^{2} \end{array}$$ (31)

$${\left\|\begin{array}{c} 2P_{ij}\\ 2Q_{ij}\\ {\tilde{I}}_{ij}-{\tilde{V}}_{i} \end{array}\right\|}_{2}\leq {\tilde{I}}_{ij}+{\tilde{V}}_{i}$$ (32)

Here, ${\tilde{I}}_{ij}$ is the current square of branch *ij*; ${\tilde{V}}_{i}$ is the voltage square at bus *i*.

In the distribution network, the injection active and reactive power for each bus are formulated as follows:

$$\{\begin{array}{l} \begin{array}{l} P_{i,t,s}=P_{t,s}^{\text{TN}}+\beta_{d,i,t,s}^{\mathrm{BESS}}P_{d,i,t,s}^{\mathrm{BESS}}+P_{i,t,s}^{\mathrm{WT}}+P_{i,t,s}^{\mathrm{PV}}\\ \;-P_{i,t,s}^{\text{WT,cur}}-P_{i,t,s}^{\text{Pv,cur}}-\beta_{c,i,t,s}^{\mathrm{BESS}}P_{c,i,t,s}^{\mathrm{BESS}}-P_{i,t}^{\mathrm{load}} \end{array}\\ Q_{i,t,s}=Q_{t,s}^{\text{TN}}+Q_{i,t,s}^{\mathrm{WT}}+Q_{i,t,s}^{\text{CBs}}\text{+}Q_{i,t,s}^{\mathrm{BESS}}-Q_{i,t}^{\mathrm{load}} \end{array}$$ (33)

Here, $P_{i,t}^{\mathrm{load}}$, $Q_{i,t}^{\mathrm{load}}$ are the active and reactive power of load for bus *i* at time *t*, respectively.

In order to ensure the safe and stable operation of distribution network, the voltage and current should meet the following constraints:

$$V_{i,\min}^{2}\leq {\tilde{V}}_{i}\leq V_{i,\max}^{2}$$ (34)

$$I_{ij,\min}^{2}\leq {\tilde{I}}_{ij}\leq I_{ij,\max}^{2}$$ (35)

Here, $V_{i,\min}^{2}$, $V_{i,\max}^{2}$ are the minimum and maximum permissible values of the voltage square at bus *i*, respectively; $I_{ij,\min}^{2}$, $I_{ij,\max}^{2}$ are the minimum and maximum permissible values of the current square of branch *ij*, respectively.

## Solution method

### The construction of worst-case scenarios

In the BESS capacity allocation model considering the uncertainty of renewable energy, a method of generating uncertainty set based on historical data proposed in [18] is adopted. The uncertainty set is a linear generalized convex hull with characteristics of spatiotemporal correlation. The specific steps to obtain this convex hull are as follows: firstly, the region consists of historical data set is fitted into a high-dimensional ellipsoid with the existing algorithms [20], after that, the coordinate transformation and the scaling of the endpoint coordinate values are carried out to transform the high-dimensional ellipsoid into a polyhedron convex hull.

**Fig 1:**
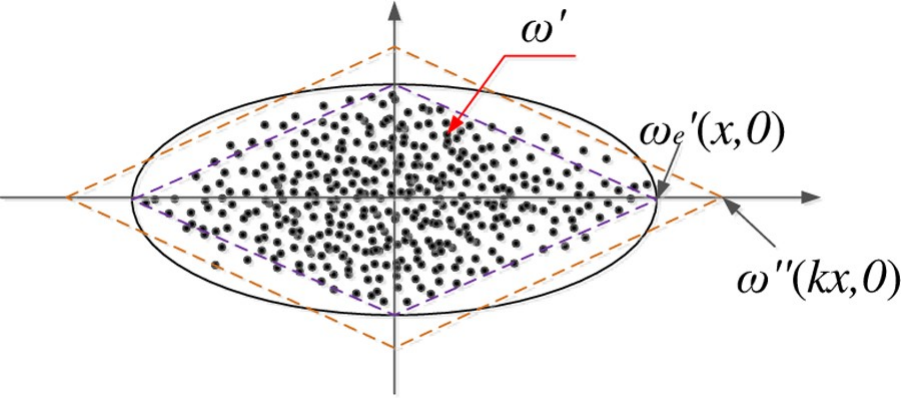
Standard ellipsoid based on historical data set.

As shown in Fig 1, the standard ellipsoid can be obtained by coordinate transformation of high-dimensional ellipsoid based on historical data set, formulated as follows:

$$E\left(W\right)=\{\omega '\in R^{gT}{|\omega '}^{T}W\omega '\leq 1\}$$ (36)

ω'=P×(ω−a) (37)

$$\omega_{n}={\left(P_{1,1}^{n}\ldots P_{1,T}^{n}\ldots P_{g,T}^{n}\right)}^{T}$$ (38)

$$\left[\omega {'}_{e,1},\cdots ,\omega {'}_{e,m}\right]=\pm diag\left(\frac{1}{\sqrt{\lambda_{1}}},\cdots ,\frac{1}{\sqrt{\lambda_{gT}}}\right)$$ (39)

Here, *E*(*W*) is standard ellipsoid after the coordinate transformation; *g* is the total number of wind farms and photovoltaic plant; *W* is a diagonal matrix; *P* is an orthogonal matrix used for the coordinate transformation; *ω*’ is a vector denoting the endpoint of standard ellipsoid; *ω*_*n*_ is the *n*th historical scenario with spatiotemporal correlation that belongs to data set *ω*; *ω*’_*e*,*m*_ is the coordinate value of the *m*th vertex of the standard ellipsoid; *λ*_*gT*_ is the *gT*th element on the diagonal of matrix W.

According to the diagonal matrix *W* in the standard ellipsoid Eq (36), the endpoints *ω*’_*e*_ of the ellipsoid on each axis can be obtained from (39). Then, the polyhedron convex hull can be enlarged or shrunk to cover all historical scenarios by using the scaling factor. An optimization model to calculate the scaling factor can be found in [18]. The expanded convex hull can be formulated as follows:

$$E\text{''}=\left\{\omega \text{''}\in R^{gT}|\begin{array}{c} \omega \text{''}=k\sum_{m=1}^{N_{m}}\delta_{m}\cdot \omega {\text{'}}_{e,m}\\ \sum_{m=1}^{N_{m}}\delta_{m}=1,\delta_{m}\geq 0 \end{array}\right\}$$ (40)

Here, *E*” is the expanded polyhedron convex hull; *ω*” is the endpoint of expanded polyhedron convex hull; *k* is a scaling factor; *N*_*m*_ is the number of total scenarios; *δ*_*m*_ is a binary variable.


$$\omega_{e,m}=P^{-1}k\omega {\text{'}}_{e,m}+a$$ (41)

The end points of the expanded convex hull are transformed back to the original historical scenario set. Accordingly, the extreme scenarios *ω*_*e*,*m*_ can be obtained by Eq (41), which are conducive to making the robust decision for the BESS configuration.

### Linearization method

Eqs (12), (13) and (33) of the above model contain the multiplication of binary variables and continuous variables. These terms need to be linearized to solve the model effectively. As a result, $\beta_{c,i,t-1,s}^{\mathrm{BESS}}P_{c,i,t,s}^{\mathrm{BESS}}$ and $\beta_{d,i,t-1,s}^{\mathrm{BESS}}P_{d,i,t,s}^{\mathrm{BESS}}$ can be equivalent to $G_{c,i,t,s}^{BESS}$ and $G_{d,i,t,s}^{BESS}$, respectively. The big-M method is used to linearize these terms as follows:

$$-\beta_{c,i,t,s}^{BESS}M\leq G_{c,i,t,s}^{BESS}\leq \beta_{c,i,t,s}^{BESS}M$$ (42)

$$-\beta_{d,i,t,s}^{BESS}M\leq G_{d,i,t,s}^{BESS}\leq \beta_{d,i,t,s}^{BESS}M$$ (43)

$$P_{c,i,t,s}^{BESS}-\left(1-\beta_{c,i,t,s}^{BESS}\right)M\leq G_{c,i,t,s}^{BESS}\leq P_{c,i,t,s}^{BESS}+\left(1-\beta_{c,i,t,s}^{BESS}\right)M$$ (44)

$$P_{d,i,t,s}^{BESS}-\left(1-\beta_{d,i,t,s}^{BESS}\right)M\leq G_{d,i,t,s}^{BESS}\leq P_{d,i,t,s}^{BESS}+\left(1-\beta_{d,i,t,s}^{BESS}\right)M$$ (45)

Here, *M* is a large constant.

### Case studies

The formulated BESS configuration model in this paper can be solved by CPLEX solver on a Core i5-9500 CPU and 8.00GB RAM desktop computer.

### System parameters description

**Fig 2:**
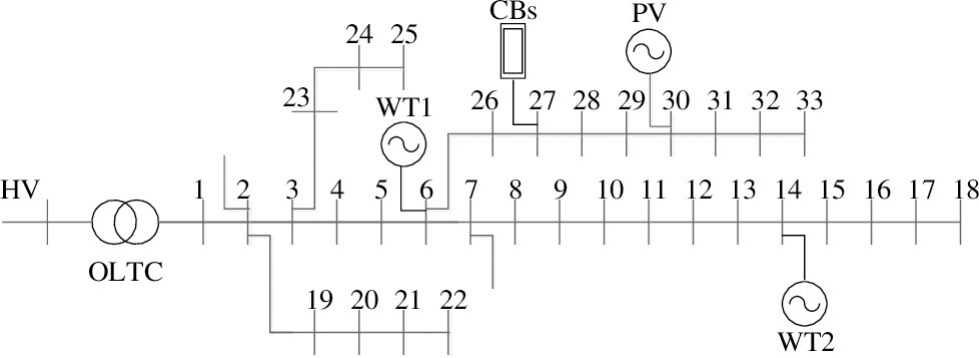
33-node active distribution network.

**Fig 3:**
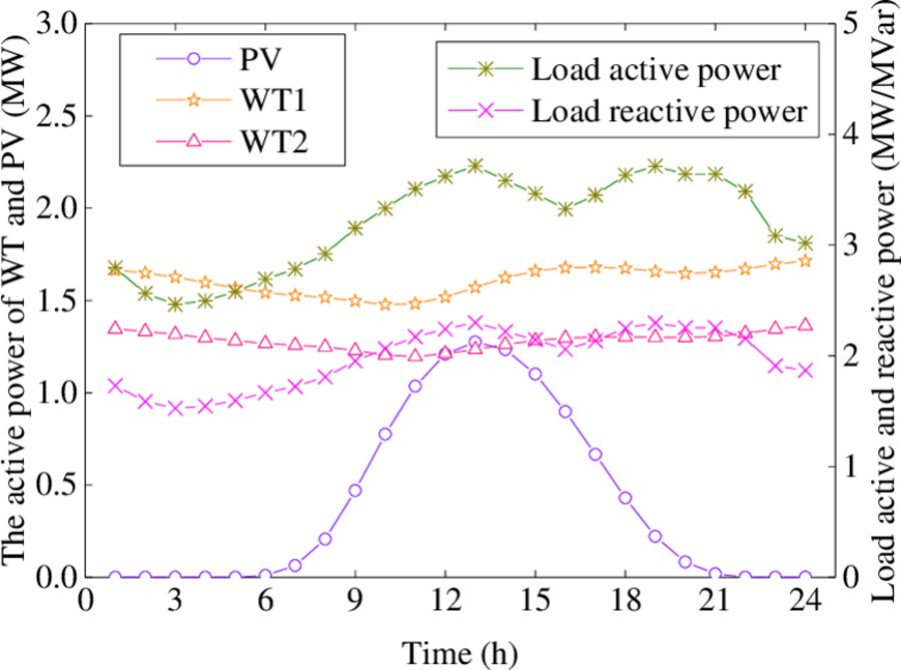
Load and renewable energy output curves.

In this paper, the modified IEEE-33 bus system, as shown in Fig 2, is employed to illustrate the performance of the proposed approach. The system topological data can be obtained from [21]. The forecast load power curves, wind and photovoltaic power profiles in a day are shown in Fig 3. The transformation ratio range of OLTC is 1±6%, and the step of its tap ratio is 0.01%. The switchable CBs is connected to bus 27, whose capacities are [0.0.6]MVar and the step is 0.06 MVar. The maximum allowable operation times over 24 hours for OLTC and CBs are 5. The upper/lower bound of WT reactive power output is ±1.2MVar. The rated voltage of the distribution network is 12.66kV, and the tolerant range of each bus voltage is [0.94,1.06], the reference voltage is 1. The current limit of each branch is 0.4kA. The lower and upper bounds of the active power from the TN are 0MW and 2.5MW, respectively. The unit price of power loss is 560 $/MWh. The penalty price of wind and photovoltaic power curtailed is 500$/MWh. The electricity prices of the TN are denoted Table 1. The wind and photovoltaic power historical data are from the literature [22]. The specific parameters about BESS are represented in Table 2.

**Table 1:** Electricity prices of main grid.

Time	Electricity prices
1-6h and 23-24h	150$/MWh
7-9h and 18-22h	400$/MWh
10-17h	250$/MWh

**Table 2:** Parameters of BESS.

Parameters	Value	Parameters	Value
*κ*	0.2	$S_{i,\max}^{\text{BESS}}$	1.5MVA
*ς*	20	$E_{i,\max}^{\mathrm{BESS}}$	2.5MWh
*μ*_*S*_	50000$ /MVA	D	0.8
*μ*_*E*_	100000$/ MWh	*η*	0.9
*μ*_*m*_	0.04		

### Case study

The BESS are installed in the wind farms and photovoltaic plant as shown in Fig 3, respectively. In order to verify the superiority of the proposed model and method, the following three cases are set up for analysis.

Case 1: the average value of the historical data is selected as a typical daily scenario to obtain the active and reactive power coordinated optimization strategy, where the BESS is not installed in the distribution network;

Case 2: the average value of the historical data is selected as a typical daily scenario. On this basis, the BESS capacity configuration scheme and the active and reactive power coordinated optimization strategy can be obtained;

Case 3: the scenarios derived from k-means clustering of historical data are selected as typical daily scenarios. On this basis, the BESS capacity configuration scheme and the active and reactive power coordinated optimization strategy can be obtained.

Case 4: the extreme scenarios considering temporal and spatial correlation are selected as typical daily scenarios. On this basis, the BESS capacity configuration scheme and the active and reactive power coordinated optimization strategy can be obtained.

It can be seen from Table 3 that the renewable energy curtailments happened in Case 1 because of the limited transmission capacity. However, in Case 2, Case 3, and Case4, the BESS installed in the distribution network can effectively enhance the capacity of wind and photovoltaic power. Besides, the power purchase cost of the TN can be reduced by charging and discharging power in an orderly manner under the guidance of time-of-use price. As the flexible device of power adjustment, the BESS can contribute to the power loss decreased of the distribution network, as well. Therefore, the actions of the BESS have a significant benefit for the economic operation. The total cost in Case 4 is higher than that of Case 2 and Case 3, the reason is that the electricity amount from TN and the investment of BESS are increased to mitigate the impact of extreme scenarios on the distribution network.

**Table 3:** Comparison of total costs in different cases.

	Case 1	Case 2	Case3	Case 4
Total cost ($)	5339.10	2963.36	3554.09	3823.01
Penalty cost of power loss ($)	2704.50	2226.83	2007.65	1978.56
Electricity cost of main grid ($)	1638.45	393.79	1140.23	1412.35
Penalty cost of wind and photovoltaic power curtailed ($)	996.15	0	0	0
Investment cost of BESS ($)	—	342.74	373.13	432.10

Comparing Case 2 to Case 3 in Table 4, the energy and power capacities of BESS installed at buses 6 and 30 in Case 3 are larger. The result shows that the scenarios based on the K-means method are more conducive to obtain a robust configuration scheme than the average value of the historical data, however, which is just an approximate representation of the characteristics of historical data. The comparison between Case 2 and Case 4 illustrates that the total cost in Case 4 is higher since the extreme scenarios set is robust for all scenarios. Therefore, the BESS with a certain capacity and power margin can stabilize the strong fluctuation of wind power and photovoltaic power by its flexible operation characteristics. In addition, due to the larger fluctuation of photovoltaic power than wind power, the power capacity of BESS installed at 30 bus is larger than that at bus 6 and 14.

**Table 4:** Configuration results of BESS.

Installation position	Power (MVA)			Capacity (MWh)		
Case 2, Case 3, Case4	Case 2	Case 3	Case 4	Case 2	Case 3	Case 4
6	0.413	0.482	0.623	2.500	2.500	2.500
14	0.534	0.520	0.495	0.429	0.863	1.437
30	0.886	0.913	0.963	2.068	2.125	2.500

**Fig 4:**
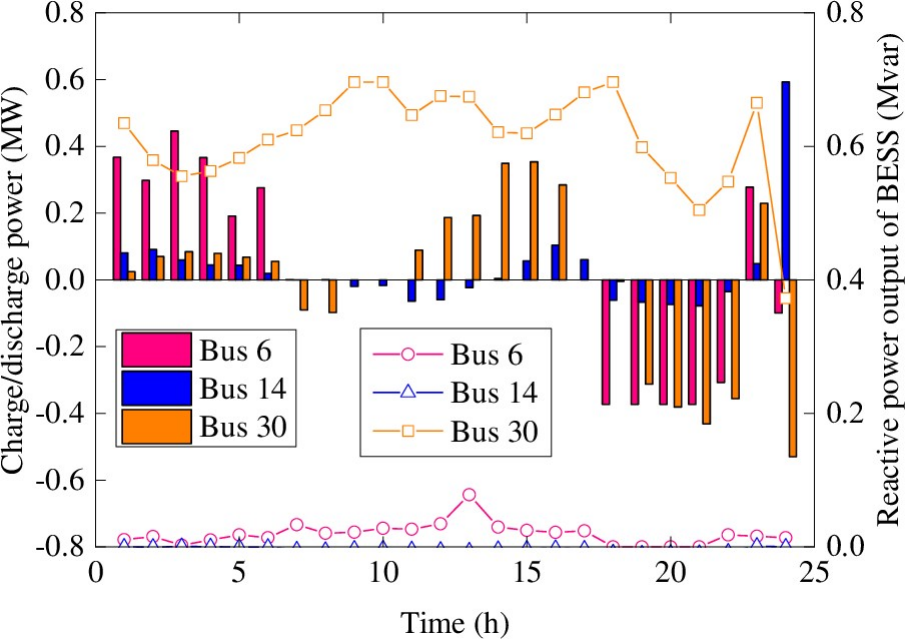
The charge and discharge power of BESS in Case 2.

As shown in Fig 4, the operation strategies of the BESS in Case 2 are that charging in the period of low electricity price and discharging in the period of peak load. To improve the economic operation of the distribution network, time-of-use electricity prices can effectively guide the charging and discharging behaviors of BESS. The BESS installed at the 30 bus of photovoltaic plant generates a larger amount of reactive power than the BESS installed at the 6 bus of a wind farm. The 14 bus wind farm generates almost hardly reactive power. As a result, the BESS can provide reactive power support for the photovoltaic power station that cannot generate reactive power. On the contrary, wind farms with a large capacity of reactive power output can ensure sufficient reactive power supply for the distribution network.

**Fig 5:**
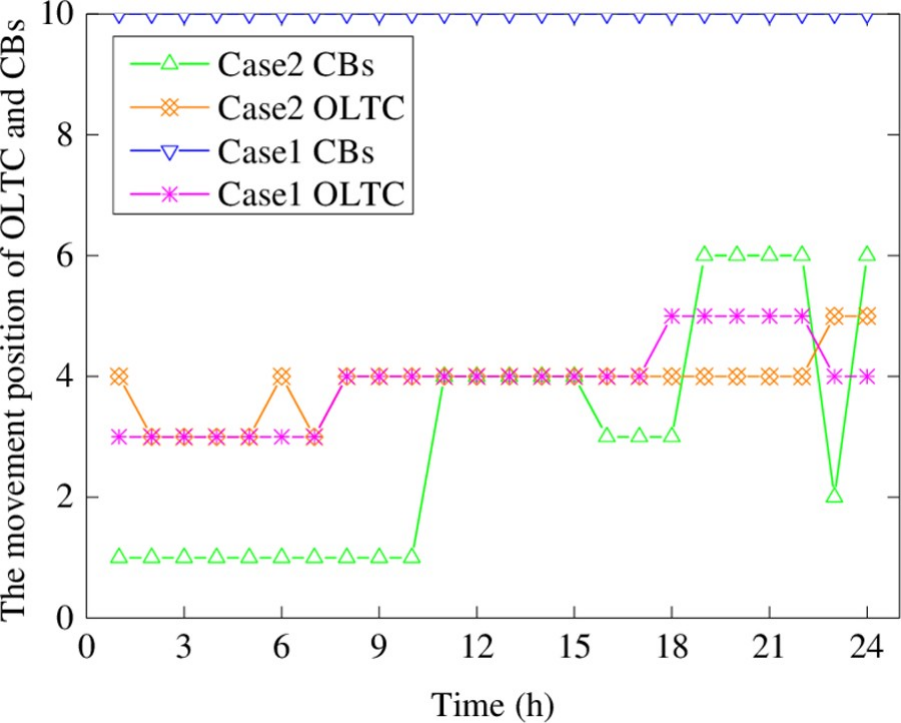
The movement position of OLTC and CBs.

It can be seen from Fig 5 that, in the whole scheduling period, the CBs position in Case 1 is in the maximum gear, while that in Case 2 can adjust reasonably according to the actual operation demand of the distribution network. From the analysis for the reactive power demand, there are fewer reactive power supply devices for the distribution network in Case 1. In addition, the OLTC gear in Case 1 is larger than that in Case 2, which contributes to the increase of electricity cost of the main grid as presented in Table 3. Therefore, when the internal energy supply of the distribution network is insufficient, the voltage at the root bus needs to be raised to facilitate the transmission of power flow. In Case 2, the load power can be supplied by discharging power to reduce the power purchase from the TN.

**Fig 6:**
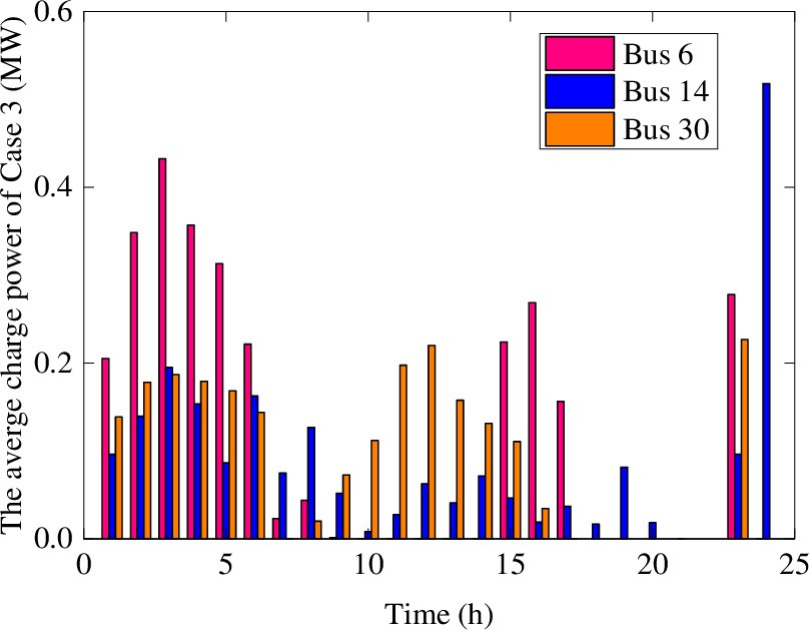
The average charging power of BESS in Case 4.

**Fig 7:**
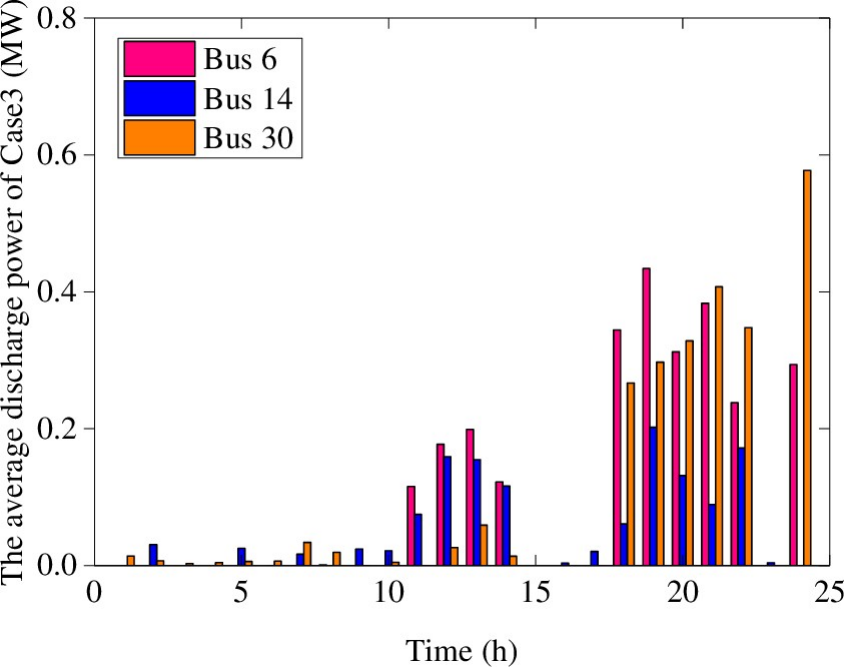
The average discharging power of BESS in Case 4.

Figs 6 and 7 show the average charging and discharging power of BESS in Case 4. The main performance is that the BESS in the wind farms charges in the initial period of the day. The BESS in the photovoltaic plant is in charging at noon and discharging happened in the evening of load peak. Compared with Fig 4, the charging and discharging period of BESS in Case 4 are more flexible, and the charging and discharging power values are versatile. In order to handle the impact of extreme scenarios with different fluctuations, the BESS with a certain margin of power and capacity can reasonably arrange the charging and discharging plans within a day. These flexible charging and discharging strategies can ensure the balance between supply and demand sides, and maintain the safe and stable operation of the distribution network.

The effectiveness of each case to improve the system voltage can be reflected by the values of total voltage deviation as follows:

$$\Delta V_{\mathrm{total}}=\sum_{t\in T}\sum_{i\in \Omega^{DN}}\frac{\left|V_{i,t}-V_{0}\right|}{V_{0}}$$ (46)

Here, Δ*V*_total_ is total voltage deviation, *V*_*i*,*t*_ is the voltage for bus *i* at time *t*, *V*_0_ is the reference voltage.

**Fig 8:**
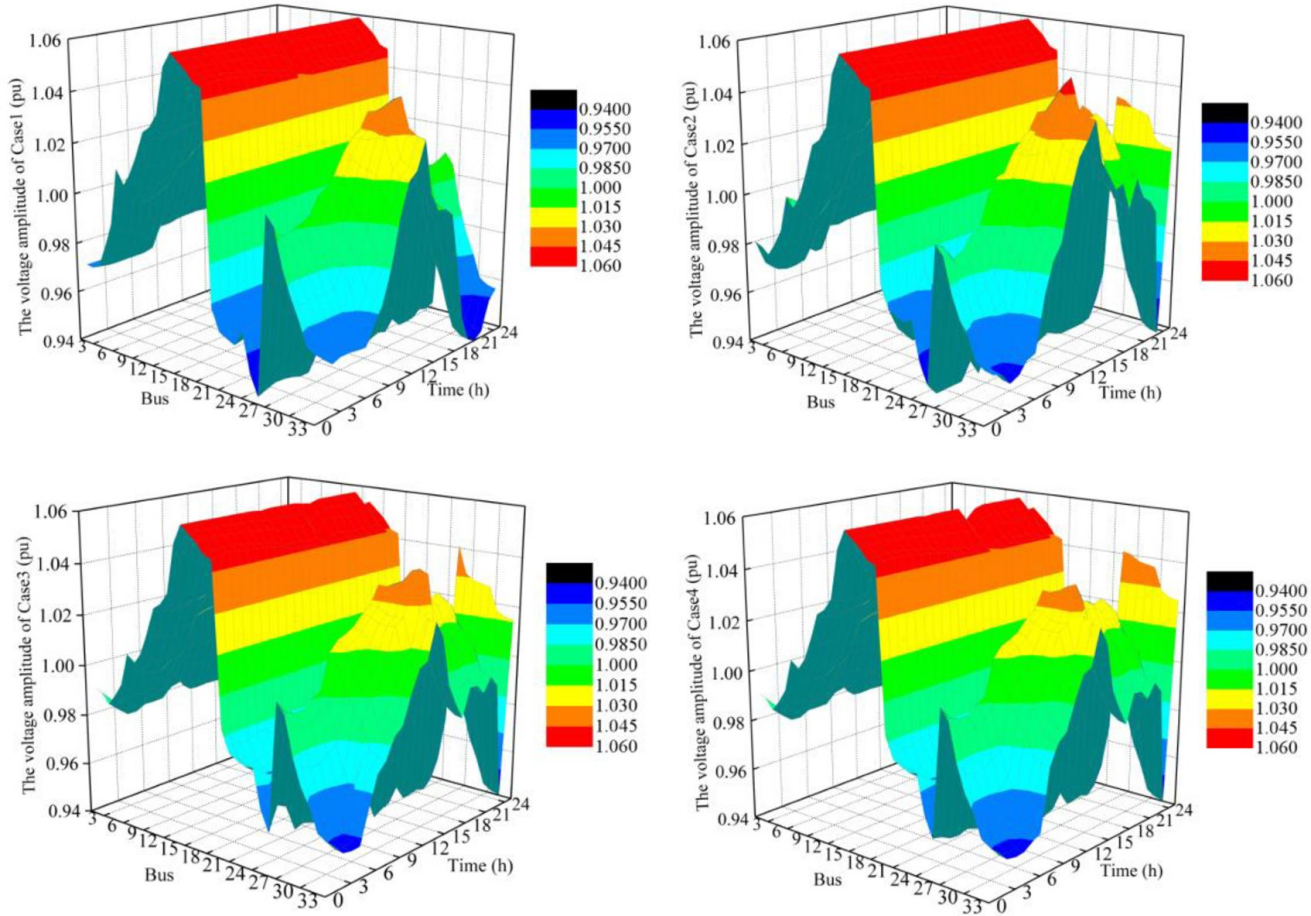
Voltage profiles in different cases.

Comparing Case 1 to other cases in Table 5, it is clear that BESS can improve the voltage quality of the distribution network. Although extreme scenarios are considered in Case 4, the BESS with four-quadrant operation characteristics also has a better voltage stability effect than Case 1. The voltage distribution in each case is shown in Fig 8. For those buses from 13 to 18, the voltage amplitude in each case is near the upper limit of voltage. For those buses from 30 to 33, the voltage values in Case 2, Case 3, and Case 4 are higher than those in Case 1 after 18 hours. As a result, the configuration of BESS in the wind farms and photovoltaic plants can effectively increase the voltage values of the terminal feeder.

**Table 5:** Comparison of total voltage deviation in different cases.

	Case 1	Case 2	Case 3	Case 4
Total voltage deviation (pu)	27.21	24.74	25.04	25.46

**Fig 9:**
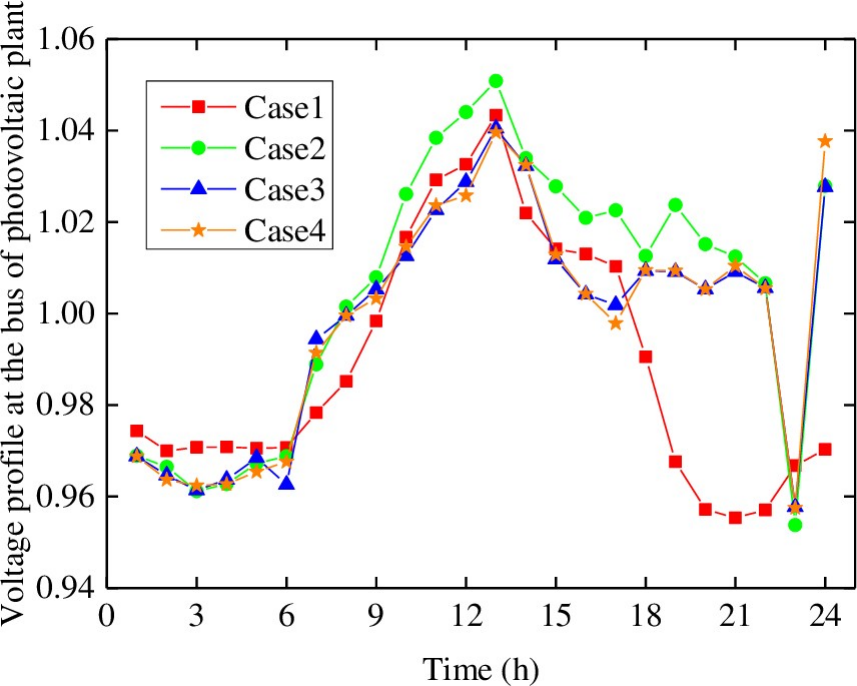
Voltage profiles at the bus of photovoltaic plant in different cases.

To further verify the advantages of each case from the perspective of reactive power, the bus voltage profile of a photovoltaic plant without a reactive power source is analyzed as shown in Fig 9. The voltage amplitude in Case 1 is small, while that in Case 2 is greatly improved after installing the BESS as well in Case 3 and Case4. The larger bus voltage is conducive to sending photovoltaic power out for the end-user, which could increase the photovoltaic power accommodation capacity of the power grid.

## Conclusions

For reducing the impact of wind power and photovoltaic power fluctuation on the operation of distribution network, this paper proposes the capacity allocation of BESS in the wind farm and photovoltaic plant from the perspective of active and reactive power coordinated optimization. The conclusions are summarized as follows:

1. The BESS with four quadrant operation characteristics is coordinated with other equipment to realize the coordinated optimization of active and reactive power in the distribution network. The active power from the TN, wind and photovoltaic power curtailment, and power loss are reduced to remarkably increase the economic benefit of the distribution network. In addition, the BESS can provide reactive power support for the distribution network, which can decrease voltage fluctuation and improve power quality of the distribution network.
2. The BESS with a certain power and capacity margin has a strong ability to suppress wind power and photovoltaic power fluctuations. Considering the extreme scenarios with temporal and spatial correlation, the robust capacity configuration decision obtained of BESS can ensure the safe and stable operation of distribution system.

As a preliminary attempt to research the BESS configuration model from the perspective of active and reactive power optimization, the uncertainties of active power outputs are considered at each scheduling period. In further research, the multi-time scale framework should be incorporated into the proposed model for more fine and accurate decisions. In addition, the uncertainties of reactive power outputs could be considered for inverter in wind farms or photovoltaic plants, since the active power fluctuation could impact the reactive power outputs of the inverter.

## Supporting information

S1 File (DOCX)
