# Supplementary material for: Configuration method of BESS in the wind farm and photovoltaic plant considering active and reactive power coordinated optimization
Source: PLoS One. 2021 Oct 13;16(10):e0257885. doi: 10.1371/journal.pone.0257885 (PMC8513916; doi:10.1371/journal.pone.0257885)
Supplement: S1 File — (DOCX) [file pone.0257885.s001.docx]

All data generated or analyzed during this study are included in this article. To carry out simulation successfully, we add the necessary data in addition to the data given in the manuscript, which can be considered as our minimal data set.

PV 0.000 0.000 0.000 0.000 0.000 0.009 0.063 0.206 0.469 0.776 1.035 1.209 1.274 1.234 1.101 0.897 0.666 0.429 0.221 0.083 0.018 0.000 0.000 0.000

WT1 1.665 1.649 1.626 1.597 1.567 1.542 1.527 1.516 1.497 1.477 1.482 1.518 1.571 1.623 1.660 1.677 1.679 1.674 1.657 1.646 1.652 1.670 1.697 1.713

WT2 1.345 1.332 1.316 1.298 1.282 1.267 1.257 1.248 1.228 1.204 1.195 1.211 1.235 1.262 1.283 1.296 1.302 1.302 1.300 1.299 1.305 1.321 1.345 1.362

Load active power profile 2.795 2.564 2.465 2.496 2.578 2.692 2.782 2.921 3.153 3.332 3.507 3.622 3.715 3.583 3.465 3.326 3.451 3.632 3.712 3.641 3.640 3.484 3.084 3.018

Load reactive power profile 1.730 1.587 1.526 1.545 1.596 1.667 1.723 1.808 1.952 2.063 2.171 2.242 2.300 2.219 2.145 2.059 2.137 2.248 2.298 2.254 2.254 2.157 1.909 1.869

The above data set corresponds to Figure 3 in the file of plos one figure (revised). (Unit: MW/Mvar)

0.000 0.000 0.000 0.000 0.000 0.000 0.000 0.000 0.000 0.000 0.000 0.000 0.000 0.000 0.000 0.000 0.000 0.000 0.000 0.000 0.000 0.000 0.000 0.000

0.075 0.069 0.066 0.067 0.069 0.072 0.075 0.079 0.085 0.090 0.094 0.097 0.100 0.096 0.093 0.090 0.093 0.098 0.100 0.098 0.098 0.094 0.083 0.081

0.068 0.062 0.060 0.060 0.062 0.065 0.067 0.071 0.076 0.081 0.085 0.088 0.090 0.087 0.084 0.081 0.084 0.088 0.090 0.088 0.088 0.084 0.075 0.073

0.090 0.083 0.080 0.081 0.083 0.087 0.090 0.094 0.102 0.108 0.113 0.117 0.120 0.116 0.112 0.107 0.111 0.117 0.120 0.118 0.118 0.113 0.100 0.097

0.045 0.041 0.040 0.040 0.042 0.043 0.045 0.047 0.051 0.054 0.057 0.058 0.060 0.058 0.056 0.054 0.056 0.059 0.060 0.059 0.059 0.056 0.050 0.049

0.045 0.041 0.040 0.040 0.042 0.043 0.045 0.047 0.051 0.054 0.057 0.058 0.060 0.058 0.056 0.054 0.056 0.059 0.060 0.059 0.059 0.056 0.050 0.049

0.150 0.138 0.133 0.134 0.139 0.145 0.150 0.157 0.170 0.179 0.189 0.195 0.200 0.193 0.187 0.179 0.186 0.196 0.200 0.196 0.196 0.188 0.166 0.162

0.150 0.138 0.133 0.134 0.139 0.145 0.150 0.157 0.170 0.179 0.189 0.195 0.200 0.193 0.187 0.179 0.186 0.196 0.200 0.196 0.196 0.188 0.166 0.162

0.045 0.041 0.040 0.040 0.042 0.043 0.045 0.047 0.051 0.054 0.057 0.058 0.060 0.058 0.056 0.054 0.056 0.059 0.060 0.059 0.059 0.056 0.050 0.049

0.045 0.041 0.040 0.040 0.042 0.043 0.045 0.047 0.051 0.054 0.057 0.058 0.060 0.058 0.056 0.054 0.056 0.059 0.060 0.059 0.059 0.056 0.050 0.049

0.034 0.031 0.030 0.030 0.031 0.033 0.034 0.035 0.038 0.040 0.042 0.044 0.045 0.043 0.042 0.040 0.042 0.044 0.045 0.044 0.044 0.042 0.037 0.037

0.045 0.041 0.040 0.040 0.042 0.043 0.045 0.047 0.051 0.054 0.057 0.058 0.060 0.058 0.056 0.054 0.056 0.059 0.060 0.059 0.059 0.056 0.050 0.049

0.045 0.041 0.040 0.040 0.042 0.043 0.045 0.047 0.051 0.054 0.057 0.058 0.060 0.058 0.056 0.054 0.056 0.059 0.060 0.059 0.059 0.056 0.050 0.049

0.090 0.083 0.080 0.081 0.083 0.087 0.090 0.094 0.102 0.108 0.113 0.117 0.120 0.116 0.112 0.107 0.111 0.117 0.120 0.118 0.118 0.113 0.100 0.097

0.045 0.041 0.040 0.040 0.042 0.043 0.045 0.047 0.051 0.054 0.057 0.058 0.060 0.058 0.056 0.054 0.056 0.059 0.060 0.059 0.059 0.056 0.050 0.049

0.045 0.041 0.040 0.040 0.042 0.043 0.045 0.047 0.051 0.054 0.057 0.058 0.060 0.058 0.056 0.054 0.056 0.059 0.060 0.059 0.059 0.056 0.050 0.049

0.045 0.041 0.040 0.040 0.042 0.043 0.045 0.047 0.051 0.054 0.057 0.058 0.060 0.058 0.056 0.054 0.056 0.059 0.060 0.059 0.059 0.056 0.050 0.049

0.068 0.062 0.060 0.060 0.062 0.065 0.067 0.071 0.076 0.081 0.085 0.088 0.090 0.087 0.084 0.081 0.084 0.088 0.090 0.088 0.088 0.084 0.075 0.073

0.068 0.062 0.060 0.060 0.062 0.065 0.067 0.071 0.076 0.081 0.085 0.088 0.090 0.087 0.084 0.081 0.084 0.088 0.090 0.088 0.088 0.084 0.075 0.073

0.068 0.062 0.060 0.060 0.062 0.065 0.067 0.071 0.076 0.081 0.085 0.088 0.090 0.087 0.084 0.081 0.084 0.088 0.090 0.088 0.088 0.084 0.075 0.073

0.068 0.062 0.060 0.060 0.062 0.065 0.067 0.071 0.076 0.081 0.085 0.088 0.090 0.087 0.084 0.081 0.084 0.088 0.090 0.088 0.088 0.084 0.075 0.073

0.068 0.062 0.060 0.060 0.062 0.065 0.067 0.071 0.076 0.081 0.085 0.088 0.090 0.087 0.084 0.081 0.084 0.088 0.090 0.088 0.088 0.084 0.075 0.073

0.068 0.062 0.060 0.060 0.062 0.065 0.067 0.071 0.076 0.081 0.085 0.088 0.090 0.087 0.084 0.081 0.084 0.088 0.090 0.088 0.088 0.084 0.075 0.073

0.316 0.290 0.279 0.282 0.291 0.304 0.315 0.330 0.356 0.377 0.396 0.409 0.420 0.405 0.392 0.376 0.390 0.411 0.420 0.412 0.412 0.394 0.349 0.341

0.316 0.290 0.279 0.282 0.291 0.304 0.315 0.330 0.356 0.377 0.396 0.409 0.420 0.405 0.392 0.376 0.390 0.411 0.420 0.412 0.412 0.394 0.349 0.341

0.045 0.041 0.040 0.040 0.042 0.043 0.045 0.047 0.051 0.054 0.057 0.058 0.060 0.058 0.056 0.054 0.056 0.059 0.060 0.059 0.059 0.056 0.050 0.049

0.045 0.041 0.040 0.040 0.042 0.043 0.045 0.047 0.051 0.054 0.057 0.058 0.060 0.058 0.056 0.054 0.056 0.059 0.060 0.059 0.059 0.056 0.050 0.049

0.045 0.041 0.040 0.040 0.042 0.043 0.045 0.047 0.051 0.054 0.057 0.058 0.060 0.058 0.056 0.054 0.056 0.059 0.060 0.059 0.059 0.056 0.050 0.049

0.090 0.083 0.080 0.081 0.083 0.087 0.090 0.094 0.102 0.108 0.113 0.117 0.120 0.116 0.112 0.107 0.111 0.117 0.120 0.118 0.118 0.113 0.100 0.097

0.150 0.138 0.133 0.134 0.139 0.145 0.150 0.157 0.170 0.179 0.189 0.195 0.200 0.193 0.187 0.179 0.186 0.196 0.200 0.196 0.196 0.188 0.166 0.162

0.113 0.104 0.100 0.101 0.104 0.109 0.112 0.118 0.127 0.135 0.142 0.146 0.150 0.145 0.140 0.134 0.139 0.147 0.150 0.147 0.147 0.141 0.125 0.122

0.158 0.145 0.139 0.141 0.146 0.152 0.157 0.165 0.178 0.188 0.198 0.205 0.210 0.203 0.196 0.188 0.195 0.205 0.210 0.206 0.206 0.197 0.174 0.171

0.045 0.041 0.040 0.040 0.042 0.043 0.045 0.047 0.051 0.054 0.057 0.058 0.060 0.058 0.056 0.054 0.056 0.059 0.060 0.059 0.059 0.056 0.050 0.049

The above data set is load active power at 33 bus 24 periods corresponding to the distribution network parameters of Figure 2 in the file of plos one figure (revised). (Unit: MW)

The above data set is load reactive power at 33 bus 24 periods corresponding to the distribution network parameters of Figure 2 in the file of plos one figure (revised). (Unit: Mvar)

0.000 0.000 0.000 0.000 0.000 0.000 0.000 0.000 0.000 0.000 0.000 0.000 0.000 0.000 0.000 0.000 0.000 0.000 0.000 0.000 0.000 0.000 0.000 0.000

0.045 0.041 0.040 0.040 0.042 0.043 0.045 0.047 0.051 0.054 0.057 0.058 0.060 0.058 0.056 0.054 0.056 0.059 0.060 0.059 0.059 0.056 0.050 0.049

0.030 0.028 0.027 0.027 0.028 0.029 0.030 0.031 0.034 0.036 0.038 0.039 0.040 0.039 0.037 0.036 0.037 0.039 0.040 0.039 0.039 0.038 0.033 0.032

0.060 0.055 0.053 0.054 0.056 0.058 0.060 0.063 0.068 0.072 0.076 0.078 0.080 0.077 0.075 0.072 0.074 0.078 0.080 0.078 0.078 0.075 0.066 0.065

0.023 0.021 0.020 0.020 0.021 0.022 0.022 0.024 0.025 0.027 0.028 0.029 0.030 0.029 0.028 0.027 0.028 0.029 0.030 0.029 0.029 0.028 0.025 0.024

0.015 0.014 0.013 0.013 0.014 0.014 0.015 0.016 0.017 0.018 0.019 0.019 0.020 0.019 0.019 0.018 0.019 0.020 0.020 0.020 0.020 0.019 0.017 0.016

0.075 0.069 0.066 0.067 0.069 0.072 0.075 0.079 0.085 0.090 0.094 0.097 0.100 0.096 0.093 0.090 0.093 0.098 0.100 0.098 0.098 0.094 0.083 0.081

0.075 0.069 0.066 0.067 0.069 0.072 0.075 0.079 0.085 0.090 0.094 0.097 0.100 0.096 0.093 0.090 0.093 0.098 0.100 0.098 0.098 0.094 0.083 0.081

0.015 0.014 0.013 0.013 0.014 0.014 0.015 0.016 0.017 0.018 0.019 0.019 0.020 0.019 0.019 0.018 0.019 0.020 0.020 0.020 0.020 0.019 0.017 0.016

0.015 0.014 0.013 0.013 0.014 0.014 0.015 0.016 0.017 0.018 0.019 0.019 0.020 0.019 0.019 0.018 0.019 0.020 0.020 0.020 0.020 0.019 0.017 0.016

0.023 0.021 0.020 0.020 0.021 0.022 0.022 0.024 0.025 0.027 0.028 0.029 0.030 0.029 0.028 0.027 0.028 0.029 0.030 0.029 0.029 0.028 0.025 0.024

0.026 0.024 0.023 0.024 0.024 0.025 0.026 0.028 0.030 0.031 0.033 0.034 0.035 0.034 0.033 0.031 0.033 0.034 0.035 0.034 0.034 0.033 0.029 0.028

0.026 0.024 0.023 0.024 0.024 0.025 0.026 0.028 0.030 0.031 0.033 0.034 0.035 0.034 0.033 0.031 0.033 0.034 0.035 0.034 0.034 0.033 0.029 0.028

0.060 0.055 0.053 0.054 0.056 0.058 0.060 0.063 0.068 0.072 0.076 0.078 0.080 0.077 0.075 0.072 0.074 0.078 0.080 0.078 0.078 0.075 0.066 0.065

0.008 0.007 0.007 0.007 0.007 0.007 0.007 0.008 0.008 0.009 0.009 0.010 0.010 0.010 0.009 0.009 0.009 0.010 0.010 0.010 0.010 0.009 0.008 0.008

0.015 0.014 0.013 0.013 0.014 0.014 0.015 0.016 0.017 0.018 0.019 0.019 0.020 0.019 0.019 0.018 0.019 0.020 0.020 0.020 0.020 0.019 0.017 0.016

0.015 0.014 0.013 0.013 0.014 0.014 0.015 0.016 0.017 0.018 0.019 0.019 0.020 0.019 0.019 0.018 0.019 0.020 0.020 0.020 0.020 0.019 0.017 0.016

0.030 0.028 0.027 0.027 0.028 0.029 0.030 0.031 0.034 0.036 0.038 0.039 0.040 0.039 0.037 0.036 0.037 0.039 0.040 0.039 0.039 0.038 0.033 0.032

0.030 0.028 0.027 0.027 0.028 0.029 0.030 0.031 0.034 0.036 0.038 0.039 0.040 0.039 0.037 0.036 0.037 0.039 0.040 0.039 0.039 0.038 0.033 0.032

0.030 0.028 0.027 0.027 0.028 0.029 0.030 0.031 0.034 0.036 0.038 0.039 0.040 0.039 0.037 0.036 0.037 0.039 0.040 0.039 0.039 0.038 0.033 0.032

0.030 0.028 0.027 0.027 0.028 0.029 0.030 0.031 0.034 0.036 0.038 0.039 0.040 0.039 0.037 0.036 0.037 0.039 0.040 0.039 0.039 0.038 0.033 0.032

0.030 0.028 0.027 0.027 0.028 0.029 0.030 0.031 0.034 0.036 0.038 0.039 0.040 0.039 0.037 0.036 0.037 0.039 0.040 0.039 0.039 0.038 0.033 0.032

0.038 0.035 0.033 0.034 0.035 0.036 0.037 0.039 0.042 0.045 0.047 0.049 0.050 0.048 0.047 0.045 0.046 0.049 0.050 0.049 0.049 0.047 0.042 0.041

0.150 0.138 0.133 0.134 0.139 0.145 0.150 0.157 0.170 0.179 0.189 0.195 0.200 0.193 0.187 0.179 0.186 0.196 0.200 0.196 0.196 0.188 0.166 0.162

0.150 0.138 0.133 0.134 0.139 0.145 0.150 0.157 0.170 0.179 0.189 0.195 0.200 0.193 0.187 0.179 0.186 0.196 0.200 0.196 0.196 0.188 0.166 0.162

0.019 0.017 0.017 0.017 0.017 0.018 0.019 0.020 0.021 0.022 0.024 0.024 0.025 0.024 0.023 0.022 0.023 0.024 0.025 0.025 0.024 0.023 0.021 0.020

0.019 0.017 0.017 0.017 0.017 0.018 0.019 0.020 0.021 0.022 0.024 0.024 0.025 0.024 0.023 0.022 0.023 0.024 0.025 0.025 0.024 0.023 0.021 0.020

0.015 0.014 0.013 0.013 0.014 0.014 0.015 0.016 0.017 0.018 0.019 0.019 0.020 0.019 0.019 0.018 0.019 0.020 0.020 0.020 0.020 0.019 0.017 0.016

0.053 0.048 0.046 0.047 0.049 0.051 0.052 0.055 0.059 0.063 0.066 0.068 0.070 0.068 0.065 0.063 0.065 0.068 0.070 0.069 0.069 0.066 0.058 0.057

0.451 0.414 0.398 0.403 0.416 0.435 0.449 0.472 0.509 0.538 0.566 0.585 0.600 0.579 0.560 0.537 0.557 0.587 0.599 0.588 0.588 0.563 0.498 0.487

0.053 0.048 0.046 0.047 0.049 0.051 0.052 0.055 0.059 0.063 0.066 0.068 0.070 0.068 0.065 0.063 0.065 0.068 0.070 0.069 0.069 0.066 0.058 0.057

0.075 0.069 0.066 0.067 0.069 0.072 0.075 0.079 0.085 0.090 0.094 0.097 0.100 0.096 0.093 0.090 0.093 0.098 0.100 0.098 0.098 0.094 0.083 0.081

0.030 0.028 0.027 0.027 0.028 0.029 0.030 0.031 0.034 0.036 0.038 0.039 0.040 0.039 0.037 0.036 0.037 0.039 0.040 0.039 0.039 0.038 0.033 0.032

The IEEE-33 system parameters corresponding to Figure 2 in the file of plos one figure (revised) are as below:

Branch Bus Bus r (ohm) x (H)

1 1 2 0.0922 0.047

2 2 3 0.493 0.2511

3 3 4 0.366 0.1864

4 4 5 0.3811 0.1941

5 5 6 0.819 0.707

6 6 7 0.1872 0.6188

7 7 8 0.7114 0.2351

8 8 9 1.03 0.74

9 9 10 1.044 0.74

10 10 11 0.1966 0.065

11 11 12 0.3744 0.1238

12 12 13 1.468 1.155

13 13 14 0.5416 0.7129

14 14 15 0.591 0.526

15 15 16 0.7463 0.545

16 16 17 1.289 1.721

17 17 18 0.732 0.574

18 2 19 0.164 0.1565

19 19 20 1.5042 1.3554

20 20 21 0.4095 0.4784

21 21 22 0.7089 0.9373

22 3 23 0.4512 0.3083

23 23 24 0.898 0.7091

24 24 25 0.896 0.7011

25 6 26 0.203 0.1034

26 26 27 0.2842 0.1447

27 27 28 1.059 0.9337

28 28 29 0.8042 0.7006

29 29 30 0.5075 0.2585

30 30 31 0.9744 0.963

31 31 32 0.3105 0.3619

32 32 33 0.341 0.5302
